# C. Starr Brewster, M. D., D. D. S.

**Published:** 1842-12

**Authors:** 


*C. Starr Brewster, M. D., D. D. S.*—We learn from "Galignani's Messenger," that the gentleman whose name heads this notice, has recently been created by the Emperor of Russia, a knight of the order of *St. Stanislas* and *Dentiste Honorare* of the Russian Court.

The "Norwich Courier," in noticing the above announcement, gives the following biographical sketch of Dr. Brewster.

Christopher Starr Brewster is the son of a highly respectable citizen of Norwich, a man still active among us, in vigorous old age. He is descended in the sixth generation, from one of the venerated company of pilgrims who landed from the May Flower on the Plymouth Rock; and there are those among us who think that his son, Dr. Brewster, possesses a claim to more honorable consideration in this high descent, than in any rank which the Autocrat of all the Russias has in his power to confer. We are not so scrupulously republican, however, as to attach no value to the well-merited distinction he has obtained abroad. Dr. B. has a still higher claim to respect in the fact that he is a self-made man, having had none but the ordinary advantages of education, and neither wealth nor professional patronage to smooth his upward way.

At the age of twenty-one, with no capital but a set of implements, in the use of which his own ingenuity was his sole instructor, he commenced the practice of dentistry, travelling northward into Canada. From thence he went westward, and down the Mississippi to New Orleans. After remaining there for a long time, he visited the West Indies, and returning, he went through most of our Southern cities, gaining skill and reputation at every step, till he established himself in Charleston, where he stood for some years at the head of his profession. He then removed to New York, and, after a short residence there, went to Paris, where he has become known throughout Europe. An operation which he performed about three years since attracted great attention, and was detailed in the public journals as a miracle of skill. It was the case of a lady, all the teeth of whose upper jaw were set horizontally, protruding from the face in a frightful manner. Dr. B. was successful in effecting a complete transformation, to the astonishment of all who knew any thing of this singular case of deformity. Dr. Brewster has since been constantly adding to his reputation by other striking performances, till we hear of his recent promotion without surprise. Indeed, we are seldom surprised at any eminence or distinction attained by any genuine son of New England.

